# The effect of paternal anxiety on mother-infant bonding in neonatal intensive care

**DOI:** 10.1186/s12884-023-06179-z

**Published:** 2024-01-11

**Authors:** Mark Ettenberger, Łucja Bieleninik, Andreas Størksen Stordal, Claire Ghetti

**Affiliations:** 1 Music Therapy Service, Department of Social Management, Fundación Santa Fe de Bogotá, Bogotá, Colombia; 2 Music Therapy Service, Clínica de la Mujer, Bogotá, Colombia; 3 SONO – Centro de Musicoterapia, Bogotá, Colombia; 4 Institute of Psychology, Faculty of Social Sciences, University of Gdańsk, Gdańsk, Poland; 5 GAMUT - The Grieg Academy Music Therapy Research Centre, NORCE Norwegian Research Centre AS, Bergen, Norway; 6 NORCE Technology, NORCE Norwegian Research Centre AS, Bergen, Norway; 7 Department of Mathematics, University of Bergen, Bergen, Norway; 8 Dept. of Music, GAMUT – The Grieg Academy Music Therapy Research Centre, The Grieg Academy, University of Bergen, Bergen, Norway; 9 Institute of Pedagogy and Languages, University of Applied Sciences, Elbląg, Poland

**Keywords:** Anxiety, Depression, Neonatal intensive care unit (NICU), Mothers, Fathers, Mental health

## Abstract

**Background:**

The hospitalization of a preterm infant in the NICU can lead to mental health difficulties in parents, but not much is known how paternal anxiety might affect the mother-infant relationship.

**Methods:**

This prospective cohort study is a secondary analysis investigating how paternal anxiety levels might affect maternal bonding in the NICU using the dataset of the multinational pragmatic randomized controlled trial LongSTEP. A linear mixed-effects model was used for correlations of paternal anxiety (GAD-7) and maternal bonding (PBQ) at NICU discharge, and at 6 and 12 months infant corrected age. Secondary analyses examined effects on paternal anxiety related to: site (Argentina, Colombia, Israel, Norway, and Poland), maternal depression (EPDS), infant gestational age at birth, paternal age, and type of pregnancy.

**Results:**

Paternal anxiety did not predict maternal bonding at NICU discharge (*p* = 0.096), at 6 months (*p* = 0.316), or at 12 months infant corrected age (*p* = 0.473). Secondary outcomes showed a statistically significant site effect, with higher paternal anxiety levels at the two Colombian sites at baseline (*p* = 0.014 and *p* = 0.020) and for one site at discharge (*p* = 0.012), but not for paternal age (*p* = 0.925 and *p* = 0.793), infant gestational age at birth (*p* = 0.974 and *p* = 0.686 and *p* = 0.340), or type of pregnancy (*p* = 0.381). Maternal depression predicted paternal anxiety at baseline (*p* < 0.001) and at discharge (*p* = 0.003).

**Conclusions:**

In this study, paternal anxiety did not predict maternal bonding. Paternal anxiety varied by site, indicating a need for research on potential cultural differences in manifestation of paternal anxiety. Maternal depression predicted paternal anxiety, confirming a previously reported correlation. Further research on variations in paternal mental health in the neonatal period is warranted, as well as exploration of the social contagion of mental health in preterm parents.

**Trial registration:**

ClinicalTrials.gov NCT03564184.

**Supplementary Information:**

The online version contains supplementary material available at 10.1186/s12884-023-06179-z.

## Introduction

With a global trend towards family-centered care, parental presence has improved in neonatal intensive care units (NICU) over the last decades. Simultaneously, a deeper understanding of infants’ and parents’ experiences in neonatal care has been gained. Parents often experience stress in the NICU [1, 2], but also other mental health challenges such as depression or anxiety [3–5].

While most studies on parental mental health in the NICU focus on mothers, in recent years, fathers’ experiences have increasingly been considered [6, 7]. Research indicates that fathers face similar mental health challenges as mothers, although to what degree is controversially discussed. While mothers generally confront higher levels of stress, anxiety, and depression than fathers [8–11], a recent meta-analysis concluded that younger fathers < 30 years of age, or fathers of extremely preterm infants are prone to high stress levels in the NICU [12]. However, differences in stress of fathers and mothers might also result from distinctive expectations regarding gender roles and care responsibilities [13]. Another study found that although mothers experienced higher depression levels in the NICU compared to fathers, they improved their mental health one month after discharge, while paternal depression scores remained elevated [4]. Furthermore, paternal perinatal depressive and affective disorders might differ at least partly from those of mothers, with a clinical picture including anxiety, abnormal illness behavior, anger attacks and/or addiction behavior [14]. As most traditional screening instruments for postpartum depression have been originally designed for mothers only, they might show limited suitability for fathers [14].

Qualitative studies on paternal experiences in the NICU hint at unique challenges for fathers. Fathers often experience feelings of being caught between caring for the mother and the baby simultaneously and having to choose between being with one or the other [15, 16]. This is particularly evident in cases where the mother is still hospitalized. In such situations, it is usually the father who sees the infant first in the NICU, needs to process the medical information, and must then pass on the information to the mother or other family members [17]. Such demands can produce additional stress and anxiety since a father’s initial priority is usually the mother [18]. Fathers may also have different coping mechanisms than mothers. While many fathers acknowledge the importance of communicating their worries and fears, they often do not express those in front of the mother to avoid burdening her further [18, 19]. Gatekeeping on the part of nurses and mothers might result in fathers feeling excluded from care activities or not being perceived as a competent care partner [19, 20]. Since paternity leave is typically shorter than maternity leave in many countries, most fathers are back to work while their infant is still hospitalized [21]. Being able to concentrate on work can be an important coping mechanism for fathers but might cause further concerns related to time limitations for seeing the infant or having to switch between work and supporting the mother or the infant [22]. Thus, when considering the mental health of fathers in the NICU, the complex interplay between socio-demographic factors, individual life trajectories, cultural contexts, the existence of social support networks, and differences in coping mechanisms needs to be acknowledged.

### Mutual effect of paternal and maternal mental health during the perinatal period

Mental health challenges of mothers and fathers, in particular depression during the perinatal period, seem to be correlated. This means that maternal depression can be a major predictor for paternal depression, but also vice versa [23–25]. In a Brazilian study, moderate to severe maternal postpartum depression was associated with paternal postpartum depression with an odds ratio of 8.44 [26]. In a study from Japan, Nishimura et al. [27] found an association of paternal depression with the partner’s depression, but also a correlation of paternal depression with other factors, such as economic anxiety, previous mental health consultation or a history of infertility treatment.

Paternal perinatal depression can also negatively affect mother-infant interaction. A study investigating the correlations between depression and anxiety in fathers and mothers and mother-child interaction at three months postpartum showed that paternal depression and anxiety negatively influenced mother-infant interaction [28]. On the contrary, fathers’ presence and care participation in the NICU has been found to be significantly correlated with lower postpartum depression levels in mothers [29]. A very recent meta-analysis on mental health of parents of preterm infants showed a clear intercorrelation between both depression and anxiety symptoms of mothers and fathers and particularly positive correlations between maternal and paternal depression and maternal and paternal anxiety [30]. Thus, considering paternal mental health is not only paramount for the fathers’ own well-being, but also important due to its effect on maternal mental health and mother-infant interaction.

While there is evidence of a correlation between paternal and maternal depression, how paternal mental health might affect mother-infant bonding in the NICU is largely unknown. This study aims to address this gap using data from the LongSTEP trial (Longitudinal Study of Music Therapy’s Effectiveness for Premature infants and their caregivers) including 213 preterm infants and their parents in five countries and eight hospitals. The primary objective of the current study was to determine if paternal anxiety levels during NICU stay and at discharge predict mother-infant bonding at NICU discharge and at 6-months and 12-months infant corrected age. The secondary aims were to determine which of the following factors predict paternal anxiety during NICU stay and at discharge: study site, paternal age, infant gestational age, type of pregnancy (singleton vs. multiple birth), and maternal depression.

## Method

### Study design

We report this prospective cohort study following the STROBE statement and guidelines for cohort studies [31]. The dataset used for this secondary analysis study was derived from the multinational pragmatic randomized controlled trial LongSTEP (Longitudinal Study of Music Therapy’s Effectiveness for Premature Infants and their Caregivers; ClinicalTrials.gov NCT03564184) [32]. The LongSTEP study took place at 8 hospital sites in five countries: Argentina, Colombia, Israel, Norway, and Poland; with a primary aim of evaluating the effect of music therapy intervention on mother-infant bonding. Treatment fidelity evaluations of the main trial demonstrate that intervention providers satisfactorily adhered to the guiding principles of the intervention, across countries [33]. As a pragmatic trial, the main study purposefully accommodated variations in the diverse real world-settings included in the trial, and no differences in effect were found by site for the main outcome of mother-infant bonding [34]. Treatment intervention protocol and main outcomes are published elsewhere [34, 35].

### Population

Families were enrolled between August 2018 and April 2020. From an original sample of parents of 213 prematurely born children, 206/213 (97%) completed assessment at NICU discharge, 196/206 (95%) at 6-months infant corrected age, and 181/206 (88%) at 12-months infant corrected age. Baseline characteristics can be seen in Table 1.

**Table 1:** Baseline characteristics across 4 study arms^c^

Baseline characteristic	SC//SC		MT/SC^a^		SC/MT		MT/MT	
	N	Value	N	Value	N	Value	N	Value
Gender (female)^1^	50	25(59)	51	23(45)	53	30(57)	52	25(48)
Singleton pregnancy^1^	50	33(66)	51	38(75)	53	43(81)	52	31(60)
Cesarean delivery route^1^	50	43(86)	51	39(76)	53	44(83)	52	42(81)
Birth weight (grams)^2^	50	1422.86 (428.2)[480,2190]	51	1364.04(426.3)[620,2270]	52	1424.02(464.08)[590,2440]	52	1391.27(420.94)[650,2335]
GA at birth (weeks)^2^	50	30.89(2.61)[22.86,34.71]	51	30.37(2.59)[25.29,34.29]	53	30.48(2.79)[22.86,34.57]	52	30.18(2.61)[25.43,34.14]
GA < 28 weeks^1^		6(12)		9(18)		9(17)		12(23)
GA 28–32 weeks^1^		23(46)		23(45)		19(36)		22(42)
GA 32–35 weeks^1^		21(42)		19(37)		25(47)		18(35)
PMA enrollment (weeks)^2^	50	33.11(1.69)[29.29,37.29]	51	33.32(2.34)[30.14,44]	53	33.1(1.69)[27.43,35.57]	52	32.77(2.06)[27.43,35.57]
Apgar at 5min^2^	49	8.53(1.43) [5, 10]	50	8.42(1,67) [1, 10]	53	8.68(1.09) [5, 10]	49	8.8(1.14) [5, 10]
Weight enrollment (grams)^2^	48	1582.25(390.92)[735,2730]	51	1675.49(494.89)[935,3680]	52	1640.48(372.01)[705,2528]	52	1577.38(406.5)[820,2460]
Estimated severity of IVH^1, b^	50		51		52		52	
Cranial Ultrasound not indicated		22(44)		17(33)		21(40)		27(52)
None		25(50)		27(53)		24(46)		21(40)
Grade 1–2		3(6)		5(10)		6(12)		4(8)
Grade 3–4		0(0)		2(4)		1(2)		0(0)
Maternal age^1^	50	33.4(5.58)	51	32.14(5.15)	51	32.22(5.71)	51	34.08(5.34)
Education mother(years)^1^	48	15.56(2.62) [8, 19]	50	15.08(3.85) [4, 21]	50	15.9(2.77) [6, 21]	51	16.49(2.63) [11, 22]
Civil status mother^2,3^	50		50		53		52	
Married or living together with partner		45		47		47		49
Usual work situation mother ^2,4^	50		51		53		52	
Full-time or self-employed		39(78)		34(67)		40(75)		37(71)
Paternal age^1^	47	36.57(7.14)	48	35.17(6.73)	50	34.48(5.79)	49	36.53(5.08)
Education father(years)^1^	44	14.98(3.11) [8, 22]	47	14.3(3.42) [3, 25]	49	15.29(3.42) [6, 21]	47	15.64(3.23) [7, 23]
Usual work situation father^2,4^	47		48		50		49	
Full-time or self-employed		43(91)		44(92)		49(98)		44(90)

Abbreviations: SD, standard deviation; GA, gestational age; PMA, post-menstrual age; IVH, intraventricular hemorrhage

^1^ N (%). ^2^ Mean (SD) [min, max]. ^3^**S**ame-sex parents were invited to participate in the study but none were enrolled

^4^ Others include part-time, homemaker/stay-at-home parent, student

^a^ IVH was diagnosed by cranial ultrasound and graded according to Papile et al. ^51^

^b^ MT, music therapy; SC, standard care. ^c^For 206 participants who were randomized twice

#### Inclusion criteria

Infants:

- Infants of both genders and from any ethnicity born < 35 weeks of gestation age.
- An estimated stay of > 2 weeks study enrollment.
- In the case of multiple pregnancies, only the first-born infant was included in the study, although sibling infants received the same interventions for ethical purposes.

Parents or caregivers:

- Willing to provide informed consent.
- Understanding the local language.
- Willing to to participate in a minimum of 2 (of 3) MT sessions per week during the NICU stay and in at least 5 (of 7) MT sessions post-discharge.
- Living within reasonable commuting distance from the treating NICU.

A detailed description of the inclusion and exclusion criteria is published elsewhere [32].

#### Exclusion criteria

- Parents unable to complete the intervention/questionnaires due to mental illness, cognitive impairment or lack of requisite language skills.

### Outcome measures

Postpartum Bonding Questionnaire (PBQ).

The PBQ is a widely used questionnaire evaluating the relationship of the mother with her infant. It consists of 25 items using a 6-point Likert scale (always, very often, quite often, sometimes, rarely, never) with a total score ranging from 0 to 125, with higher scores indicating a more problematic relationship [36]. In this study, the PBQ was used for mothers only.

General Anxiety Disorder Scale (GAD-7).

The GAD-7 is a 7-item self-rated questionnaire assessing anxiety [37]. A 4-point Likert scale (nearly every day, more than half of the days, several days, not at all) is used for each item, with total scores ranging from 0 to 21. Higher scores indicate a higher perceived anxiety. The GAD-7 was used for both mothers and fathers participating in the study.

Edinburgh Postnatal Depression Scale (EPDS).

The EPDS is a 10-item self-report questionnaire evaluating maternal postpartum depression [38]. It consists of 10 items rated on a 4-point Likert scale (items vary according to questions) and total scores range from 0 to 30. A higher total score indicates more depressive symptoms. The EPDS was used only for mothers in this study.

### Statistical analysis

Descriptive methods were used to describe the sample. The association of paternal anxiety with PBQ was tested using a linear mixed-effects model (ANCOVA) at three different time points (discharge, 6 months, 12 months) with site as a random effect due to stratified randomization. The secondary outcomes were analyzed with a similar linear mixed-effects model. No special approaches for missing data were implemented as the number of missing data was low to moderate for all outcomes (< 25%) and data were assumed to be missing at random.

The quality of the models was evaluated using residual plots. All models were analyzed using the software R (version 4.2.2) with two-sided 5% significance levels. Details of statistical analysis including R codes can be found in Appendix 1.

## Results

Paternal anxiety at baseline did not predict maternal bonding, with coefficients estimated at discharge − 0.11 (*p* = 0.096, CI -0.25–0.02), at 6 months infant corrected age 0.11 (*p* = 0.316, CI -0.11–0.33), and at 12 months infant corrected age − 0.09 (*p* = 0.473, CI -0.32–0.15). Likewise, paternal anxiety at discharge did not predict maternal bonding, with coefficients estimated at discharge 0.11 (*p* = 0.155, CI -0.04–0.27), at 6 months infant corrected age − 0.06 (*p* = 0.650, CI -0.32–0.20), and at 12 months infant corrected age 0.04 (*p* = 0.800, CI -0.25–0.32). Table 2 shows results from statistical analysis of the primary outcome.

**Table 2:** Results from statistical analysis of the association between paternal anxiety and bonding

*Predictors*	PBQ Discharge			PBQ 6 months			PBQ 12 months		
	*Estimates*	*CI*	*p*	*Estimates*	*CI*	*p*	*Estimates*	*CI*	*p*
**(Intercept)**	1.03	-0.07–2.13	0.067	3.63	1.54–5.72	**0.001**	4.67	2.11–7.24	**< 0.001**
**PBQ baseline**	0.61	0.53–0.69	**< 0.001**	0.41	0.28–0.54	**< 0.001**	0.36	0.22–0.50	**< 0.001**
**Paternal GAD-7 baseline**	-0.11	-0.25–0.02	0.096	0.11	-0.11–0.33	0.316	-0.09	-0.32–0.15	0.473
**Paternal GAD-7 discharge**	0.11	-0.04–0.27	0.155	-0.06	-0.32–0.20	0.650	0.04	-0.25–0.32	0.800
**Observations**	180			168			161		

Paternal age, infant gestational age at birth, and type of pregnancy (single, multiple), did not predict paternal anxiety at either baseline or discharge.

A statistically significant relationship was found for maternal depression at baseline predicting paternal anxiety at baseline (*p* < 0.001, CI 0.25–0.52) and for maternal depression at discharge predicting paternal anxiety at discharge (*p* = 0.003, CI 0.07–0.36). Additionally, there was a significant site effect with the two Colombian sites showing higher paternal anxiety levels at baseline than at the other sites (*p* = 0.014, CI 1.00–8.78, and *p* = 0.020, CI 0.56–6.58, respectively), and one showing higher paternal anxiety at discharge than other sites (*p* = 0.012, CI 0.96–7.68). Table 3 shows results from statistical analysis of the secondary outcomes.

**Table 3:** Results from statistical analysis of the association between other variables and paternal anxiety

*Predictors*	Paternal GAD-7 Baseline			Paternal GAD-7 Discharge		
	*Estimates*	*CI*	*p*	*Estimates*	*CI*	*p*
**(Intercept)**	0.07	-8.88–9.02	0.987	5.38	-2.12–12.89	0.159
**Paternal age**	-0.01	-0.12–0.11	0.925	-0.01	-0.11–0.08	0.793
**Multiple birth**	-0.32	-1.91–1.26	0.686	0.56	-0.70–1.83	0.381
**EPDS baseline**	0.39	0.25–0.52	**< 0.001**	-0.07	-0.21–0.07	0.345
**Gestational age birth**	0.00	-0.27–0.28	0.974	-0.11	-0.33–0.11	0.340
**Site [COL-1]**	4.89	1.00–8.78	**0.014**	4.32	0.96–7.68	**0.012**
**Site [COL-2]**	3.57	0.56–6.58	**0.020**	-1.63	-4.35–1.09	0.238
**Site [ISR]**	2.21	-0.73–5.14	0.140	0.41	-2.23–3.06	0.759
**Site [NOR-1]**	0.59	-4.08–5.26	0.803	-0.49	-4.35–3.37	0.802
**Site [NOR-2]**	2.14	-1.27–5.56	0.217	0.52	-2.51–3.55	0.734
**Site [NOR-3]**	2.24	-3.22–7.71	0.419	1.08	-3.88–6.04	0.668
**Site** **[POL]**	1.72	-1.28–4.73	0.260	0.43	-2.28–3.15	0.754
**Paternal GAD-7 baseline**				0.32	0.21–0.44	**< 0.001**
**EPDS discharge**				0.21	0.07–0.36	**0.003**
**Observations**	194			182		

## Discussion

In this study, no significant association between paternal anxiety and maternal bonding was found. Although both prenatal depressive and anxiety symptoms have been found to be correlated with paternal bonding postnatally [39], to our knowledge, no other study has been published that investigates how paternal anxiety in the NICU influences mother-infant bonding. However, previously reported links between maternal and paternal mental health was also confirmed in our study, in which maternal depression predicted paternal anxiety during the NICU stay. In a recent Polish study, paternal postpartum bonding was found to be correlated with maternal postpartum bonding, maternal stress, and paternal anxiety, although only parents of full-term infants were assessed [40]. The relationship between paternal and maternal mental health is paramount, since both can negatively affect parent-infant interactions and infant development [28, 41, 42]. Yet, it remains unclear if such associations continue across childhood and adolescence. A follow-up study of parental mental health at 11–12 years of child age revealed no relation of maternal anxiety and depression with paternal depression, although a small effect size for paternal anxiety was found at this time [43]. Since the data of the current study come from parents in five countries (Argentina, Colombia, Israel, Norway, and Poland), the association between maternal depression and paternal anxiety seems to be cross-cultural. Still, more subtle variations that could be caused by cultural differences need to be acknowledged. For example, fathers from the two Colombian sites showed significantly higher anxiety levels at baseline compared to the other sites. While we can only speculate about why this would be the case, social and cultural norms and expectations about communicating and expressing emotions and feelings between Colombian and European/Middle East fathers might play a role. The role of culture in the NICU has been previously addressed [44], but certainly warrants further consideration.

While in this study paternal postpartum depression was not measured, critical voices raise concerns about the suitability of applying such scales developed for mothers also to fathers [14, 17]. Postpartum depressive symptoms of fathers might vary from those of mothers and can include anxiety, abnormal illness behavior, anger, or increased substance abuse, among others [14]. In clinical practice it might therefore be important to evaluate if anxiety in fathers of preterm infants could be a sign of a more general depressive symptomatology. Pre-screening of both maternal and paternal mental health during the perinatal period is certainly indicated as part of best practice in NICUs, especially with the aim to provide additional support for parents at risk for mental health problems.

Though a recent systematic review found higher paternal stress in the NICU among fathers under 30 years of age and in those with an extremely preterm infant [12], in our study paternal age and infant gestational age at birth did not predict paternal anxiety during hospitalization. Our results might hint at differential effects of socio-demographic (e.g., available social support networks), cultural (e.g., how stress is manifested or expressed in each society), and infant-related factors on either stress or anxiety. However, this needs to be explored in future research.

Overall, the results from our study suggest that the postnatal period can be a vulnerable time also for fathers. Early parenting interventions should focus therefore on both parents, adjusted to the needs of each particular family. Considering that mothers and fathers might experience, express, and cope with mental health challenges differently, might help to provide more individually tailored support during the NICU stay.

### Strengths, limitations, and future directions

This study has several limitations. First, since primary and secondary outcomes of the main trial relied primarily on parental self-report questionnaires, this increases the risk of bias compared to clinically observed outcomes. Second, fathers were not assessed for depressive symptoms, just for general anxiety. While anxiety is an important indicator of mental health, as discussed above, high anxiety levels could also be a sign of a more general depressive symptomatology [14]. Third, while the overall sample size in this study was approximately 180 fathers (depending on the timepoint), for each country and site, the sample sizes were relatively small and might not be representative. Also, we did not measure other confounding factors affecting paternal mental health, such as the quality of social support systems, legal frameworks related to paternity leave in each country, or perinatal medical complications of the mother, just to mention a few. And lastly, the anxiety measure used in this study (GAD-7) measures general anxiety and not post-partum specific anxiety. A recently published short form (12 items) of the Postpartum Specific Anxiety Scale (PSAS) [45] might be indicated for future studies in the NICU context.

We recommend that fathers’ well-being and mental health will hopefully be given greater attention in future research and clinical practice. Little is known about which forms of support might be most favorable for fathers in the NICU, or the optimal time points, frequency, and duration of such support. On the other hand, several studies point out that most parents in the NICU cope well with increased stress during this time [13]. Thus, research on sub-groups that already show a risk during perinatal mental health screening would be indicated [34]. And finally, our results indicate that the impact of culture on mental health needs to be considered. It is known that cultural diversity affects how mental health is approached by patients, healthcare providers, and the society [46, 47]. A future study focusing on culture-dependent factors affecting mental health in NICU parents could help to reveal such aspects.

## Conclusion

To our knowledge, this is the first study investigating the association between paternal anxiety and mother-infant bonding in the NICU. While the unique experiences of fathers of preterm infants have increasingly become a target for investigation, more large-scale and cross-cultural studies on paternal mental health in this context are needed. While this study demonstrated no predictive relationship between paternal anxiety and mother-infant bonding, an association between maternal postpartum depressive symptoms and paternal anxiety levels has been confirmed. The complex interplay between cultural, socio-demographic, systemic, and biographical factors should be acknowledged when considering mental health in parents in the NICU.

### Electronic supplementary material

Below is the link to the electronic supplementary material.

Appendix 1

## Data Availability

Deidentified individual participant data will not be made available for this study, but deidentified participant data from the full data set of the main trial are available through OSF at the following URL: 10.17605/osf.io/smjka.

## Declarations

### Clinical trial registration

ClinicalTrials.gov NCT03564184, https://clinicaltrials.gov/ct2/show/NCT03564184.

## Abbreviations

NICU: Neonatal Intensive Care Unit
PBQ: Postpartum Bonding Questionnaire
GAD-7: General Anxiety Disorder scale
EPDS: Edinburgh Postnatal Depression Scale
