# Supplementary material for: The effect of paternal anxiety on mother-infant bonding in neonatal intensive care
Source: BMC Pregnancy Childbirth. 2024 Jan 11;24:55. doi: 10.1186/s12884-023-06179-z (PMC10782755; doi:10.1186/s12884-023-06179-z)
Supplement: Supplementary file 1 — Appendix 1 [file 12884_2023_6179_MOESM1_ESM.docx]

*Appendix 1*

*Statistical analysis with R codes*

Descriptive methods were used to describe the sample. The association of paternal anxiety with PBQ was tested using a linear mixed-effects model (ANCOVA) at three different time points (discharge, 6 months, 12 months) with site as a random effect due to stratified randomization (PBQ[time] ~ PBQ[baseline] + GAD7[time] + 1|site). The secondary outcomes were analyzed with a linear model (GAD7[baseline]~age + multiple birth + EPDS[baseline] + GA at birth + site) for baseline and GAD7[discharge]~age + multiple birth + GAD7[baseline] + EPDS[baseline] + EPDS[discharge] + GA at birth + site for discharge). No special approaches for missing data were implemented as the number of missing data was low to moderate for all outcomes (<25%) and data were assumed to be missing at random.
